# Neural Efficiency of Human–Robotic Feedback Modalities Under Stress Differs With Gender

**DOI:** 10.3389/fnhum.2019.00287

**Published:** 2019-08-30

**Authors:** Joseph K. Nuamah, Whitney Mantooth, Rohith Karthikeyan, Ranjana K. Mehta, Seok Chang Ryu

**Affiliations:** ^1^NeuroErgonomics Laboratory, Department of Industrial & Systems Engineering, Texas A&M University, College Station, TX, United States; ^2^Department of Environmental and Occupational Health, Texas A&M University, College Station, TX, United States; ^3^Department of Mechanical Engineering, Texas A&M University, College Station, TX, United States

**Keywords:** fNIRS, prefrontal cortex, tracking error, haptic, visual

## Abstract

Sensory feedback, which can be presented in different modalities – single and combined, aids task performance in human–robotic interaction (HRI). However, combining feedback modalities does not always lead to optimal performance. Indeed, it is not known how feedback modalities affect operator performance under stress. Furthermore, there is limited information on how feedback affects neural processes differently for males and females and under stress. This is a critical gap in the literature, particularly in the domain of surgical robotics, where surgeons are under challenging socio-technical environments that burden them physiologically. In the present study, we posited operator performance as the summation of task performance and neurophysiological cost of maintaining that performance. In a within-subject design, we used functional near-infrared spectroscopy to assess cerebral activations of 12 participants who underwent a 3D manipulation task within a virtual environment with concurrent feedback (visual and visual + haptic) in the presence and absence of a cognitive stressor. Cognitive stress was induced with the serial-7 subtraction test. We found that while task performance was higher with visual than visual + haptic feedback, it degraded under stress. The two feedback modalities were found to be associated with varying neural activities and neural efficiencies, and these were stress- and gender-dependent. Our findings engender further investigation into effectiveness of feedback modalities on males and females under stressful conditions in HRI.

## Introduction

In human–robotic interactions (HRIs), operators require feedback on the current state of the process, or the status of the process being controlled by the robot (Wickens et al., 2015) to prevent mode confusion. Feedback has been applied in many domains including human–vehicle interaction (Politis et al., 2014), prosthetic limbs (Jimenez and Fishel, 2014) and assistive devices (Park et al., 2015). Feedback in robotic surgery training and robot-assisted minimally invasive surgical operations is essential for effective learning of operative skills (McKendy et al., 2017). Feedback allows surgeons to safely maneuver and manipulate surgical tools, including robots, during surgical operations (Abiri et al., 2018; Miyazaki et al., 2019).

One approach to providing feedback to surgeons is to employ multi-modal interfaces (Wickens et al., 2015) – using multiple sensory modalities to present information (Freeman et al., 2017) based on Wicken’s Multiple Resource Theory (MRT; Wickens, 2002). However, combining feedback modalities does not always lead to optimal performance (Benz and Nitsch, 2017); poorly presented or excessive feedback can be as bad as no feedback at all (Wickens et al., 2015). What is the optimal combination of feedback modalities (e.g., visual + haptic or visual + auditory or auditory + haptic or visual + auditory + haptic)? That depends, in part, on the task type (Burke et al., 2006), individual preferences (Koehn and Kuchenbecker, 2015), individual differences (Park et al., 2012), and context (van Huysduynen et al., 2016; Adebiyi et al., 2017). In the surgical robotics domain, surgeons have been found to better characterize tissue via palpation in multi-modal (visual + haptic) feedback than in a single modality (haptic) feedback (Abiri et al., 2018). Furthermore, haptic feedback has been found to reduce the effects of visual-perceptual mismatches (Abiri et al., 2017). However, mixed and inconsistent results on the effectiveness of haptic feedback exist. For example, haptic feedback was shown to be ineffective in transferring surgical skills to medical students in a virtual simulated environment (Våpenstad et al., 2017) and failed to provide significant improvement in surgical performance (Van der Meijden and Schijven, 2009). Potential contributors to such inconsistencies may include variations between individuals in perceiving feedback effectiveness, their preferences, and the load the modalities place on different user groups.

Even though previous studies (e.g., Van Erp and Verschoor, 2004; Burke et al., 2006; Lee and Spence, 2008) suggest that multimodal feedback enhances the performance of participants performing multiple-tasks under high demanding conditions, its effectiveness under stress conditions has never been studied. Gender is known to influence cognitive (e.g., Munro et al., 2012; Castonguay et al., 2015) and physical (e.g., Hunter, 2014) task performance. Also, men and women differ in the way they cope with and handle stress (Matud, 2004; Bonneville-Roussy et al., 2017; Goldfarb et al., 2019). Men have been found to exhibit higher prefrontal cortex (PFC) responses during stress than women (Seo et al., 2011; Goldfarb et al., 2019), whereas women have showed higher responses in the limbic regions (Wang et al., 2007; Kogler et al., 2015a; Goldfarb et al., 2019). However, whether multimodal feedback is moderated by the combined effect of gender and stress has never been studied. This is critically important in the surgical robotics applications, where surgeons and residents experience high levels of acute stress, which impairs their performance and impacts overall patient safety (Jones et al., 2015; Georgiou et al., 2017).

Indeed, residents and surgeons’ performance can be influenced by the interplay of their attributes (e.g., expertise, stress reactivity; Berguer and Smith, 2006; Shafiei et al., 2018), task (e.g., novel, activities that challenge different modalities, codes, and stages; Wickens, 2002), and context (e.g., laparoscopic, robot surgery, open surgery; Smith et al., 2003). Thus, it is crucial that several dimensions of surgeon capabilities and limitations, outside of task performance, be determined to understand the surgeon’s performance envelope. It is worth noting that human performance is not equal to task performance. Our position is that human (in this case, a surgeon) performance is the summation of task performance and neurophysiological cost of maintaining that performance (see Equation 1), which is of particular importance in safety-critical scenarios like surgical operations.

(1) Surgeon⁢performance=∑(task⁢performances−neurophysiological⁢cost)

Neurophysiological cost of maintaining performance can be measured with functional near-infrared spectroscopy (fNIRS), an optical imaging technique to monitor cortical brain activation (Gateau et al., 2018). fNIRS has been used in the surgical robotics domain to evaluate the impact of expertise on a knot-tying task (Leff et al., 2008), to compare impacts of robotic and conventional laparoscopic surgeries in surgeons performing a suturing task (Singh et al., 2018), and to evaluate arrangements aiding hand-eye coordination (Miura et al., 2018). However, very limited work has been published that examined the neural cost associated with different feedback modalities, as much of the current efforts utilize task performance metrics (e.g., accuracy, speed) and/or subjective assessments of workload and preferences. Recently, Curtin and Ayaz (2019) combined measures of neural activity and task-related performance measures into a novel metric –neural efficiency, that enabled them to establish a relationship between neural activity and performance. High neural efficiency is achieved when performance (e.g., accuracy) is high and the corresponding mental effort (e.g., level of concentration of oxygenated hemoglobin) is low, and vice versa (Curtin and Ayaz, 2019). Using fNIRs, Curtin and Ayaz (2019) found neural efficiency in the dorsolateral PFC of participants engaged in symbol digital substitution task consistently increased through practice.

Design of effective surgical feedback systems should seek to not only minimize the neural cost on surgeons during complex surgeries but also enhance their performance under added stress while considering gender differences. This is particularly important as surgeons are under extremely challenging socio-technical environments that burden them both physiologically and psychologically (Park et al., 2010; Sari et al., 2010). The objectives of the present study were to determine if (1) different feedback modalities (visual or visual + haptic feedback) place distinct neural costs on users, and whether this is associated with maintenance or enhancement of task performance, and (2) neural efficiency of different feedbacks differ between males and females under stress.

## Materials and Methods

### Participants

Twelve right-handed non-smokers (six males and six females) with mean (SD) age of 25.5 (2.68) years, height of 1.71 (0.08) m, and weight of 75.46 (16.78) kg participated in the study. Participants did not have previous experience using the Novint Falcon (Novint Technologies, Inc., Albuquerque, NM, United States) gaming interface. The study was approved by the Texas A&M University Institutional Review Board.

#### Experimental Design Conditions

The main factors under study were feedback modality (visual vs. visual + haptic), cognitive stress (no computation vs. computation), and gender (male vs. female). All participants underwent all feedback and stress conditions. To induce stress, we instructed participants to perform the serial-7 subtraction task concurrently with the experimental tasks.

#### Cognitive Stress Manipulation

The serial-*n* subtraction task, part of the Trier Social Stress Task (TSST; Kirschbaum et al., 1993), has been used extensively to induce cognitive stress (Taylor et al., 2006; Ritter et al., 2007; Bershad et al., 2018; Klatzkin et al., 2018, 2019). It involves repeatedly subtracting an n digit number from a 3- or 4- digit number. In the present study, we adopted the serial-7 subtraction test, requiring participants to repeatedly subtract 7 from a 3- or a 4-digit number provided to them at the start of each 30 s trial.

#### Human–Robotic Interaction

**FIGURE 1:**
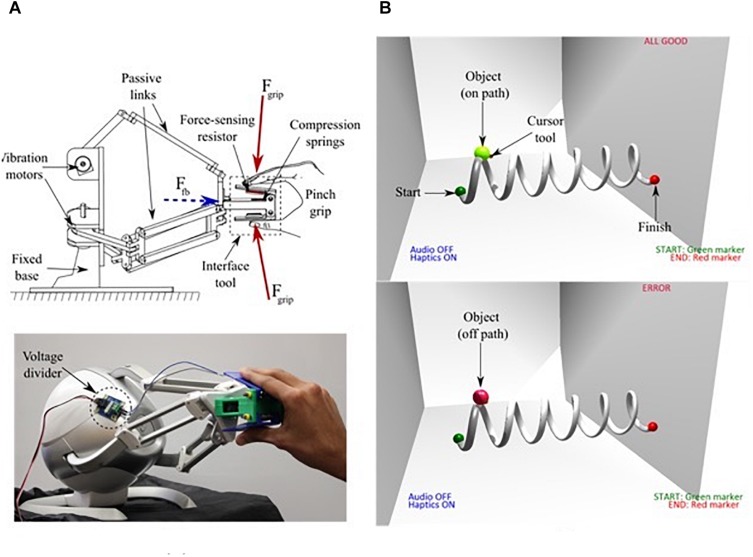
**(A)** Schematic representation of user interaction with the haptic interface showing the directions of grip force (*F*_grip_) and feedback (*F*_fb_) (top). Participant interaction with the input tool of the Novint Falcon (bottom), and **(B)** The Virtual 3D environment and task path [on path (top), off path (bottom)].

We used the Novint Falcon 3D joystick (Novint Technologies, Inc., Albuquerque, NM, United States), to implement interaction between participants and our virtual robotic system. The input tool of the 3D touch device was a 3D printed pinch grip made of polylactic acid (PLA) polymer, with box dimensions 5.04 cm × 6.04 cm × 2.13 cm, as shown in Figure 1A. It was important to ensure sufficient contact force between the user’s hand and the input tool. This ensures a 1:1 correspondence between the feedback (*F*_*fb*_) delivered at the attachment plate and the gripper. For this reason, the pinch grip consisted of a compression spring in contact with an analog force-sensing resistor (FSR) (Thin Force Sensor 1131_0, Phidgets, Inc., Canada) that monitored user contact force (*F*_*grip*_) during the experiment. The force-sensor was interfaced using an analog I/O board (1018_0 Interface Kit, Phidgets, Inc., Canada) to a Master PC that rendered a 3D virtual environment for user interaction. For the scope of this study, the FSR functioned as a binary switch that ensured sufficient contact force for object manipulation. During the experiments, the force data confirms consistent and firm contact between the gripper and fingers with an average value of *F*_*grip*_ > 20 N. Therefore, vibrotactile feedback was effectively delivered to the subjects. The Novint Falcon was the joystick that participants used to manipulate objects and traverse simple trajectories during the experiment. The visual interface was built using OpenGL and Visual C++, with the Falcon-compatible Force Dimension SDK (Force Dimension, Nyon, Switzerland), and Chai3D (SAIL, Stanford University, Stanford, CA, United States) haptic libraries. The virtual environment consisted of a three-dimensional cubic room, where the user was provided an isometric field-of-view.

#### Feedback Modalities

##### Visual feedback

We built a virtual interface using OpenGL, Visual C++, Falcon-compatible Force Dimension SDK (Force Dimension, Nyon, Switzerland), and Chai3D haptic libraries that provided participants with an isometric field-of-view of a three-dimensional cubic room (see Figure 1B). In the visual feedback condition, the object’s color changed to indicate whether the object was on or off the path.

##### Visual + haptic feedback

Participants used the Novint Falcon as the joystick to manipulate objects and traverse simple trajectories. Vibration motors located within the Falcon’s base rendered force and vibratory feedback (haptic stimuli). Passive links transduced these vibrations to the attachment plate where a 3D printed input tool (the gripper) was fixed. The three motors that actuated the arms of the parallel mechanism together generated a peak amplitude of 8.9 N at the end-effector. A software limit of 0.2 × *peak force* amplitude was set to provide feedback that did not impede user performance. In the visual + haptic feedback condition, the input tool provided vibratory feedback at 75 Hz, and peak amplitude of approximately 1.78 N when the user’s position was outside the prescribed path, along with the object color change that indicated on/off path feedback. Figure 1B shows what participants saw when an error occurs versus no error when completing the task.

### Procedures

We obtained participants’ written informed consent, after which we collected their demographic information. After bio-instrumentation with the fNIRS head cap, discussed later, we instructed participants to sit on an adjustable chair with their backs and feet firmly supported. Next, we performed three baseline measurements protocol along with task familiarization.

First, we asked participants to focus their attention on a fixed point at eye level for 10 s, and then close their eyes for 20 s. Participants repeated these three times. Second, we instructed participants to close their eyes relax, breathe normally, and refrain from any activity for 2 min. Third, we instructed participants to perform 3 maximum voluntary contractions (MVCs) using a three-point pinch grip. Participants held a one-inch thick hard block. We instructed them to squeeze as hard as they could for 5 s, followed by 2 min of rest. Then, we instructed participants to use a three-point grasp to interact with the input tool of a haptic-enabled 3D touch device (Novint Falcon), located on a height-adjustable table. Participants used this device to manipulate objects and traverse simple trajectories during the experiment. We provided participants with a 10-min time window in order for them to get familiar with the input tool and virtual environment before the start of each experiment. This included two demo applications – object shape matching tasks and trajectory tracking tasks with increasing complexity.

The main experiment consisted of four conditions: visual feedback under no stress, visual feedback under stress, visual + haptic feedback under no stress, visual + haptic feedback under stress. We counterbalanced the order of the conditions to minimize any learning effects. In each experimental condition, we instructed participants to move a spherical object from a start point to an end point along a 3D helical path within a 30 s window. We instructed them to be as precise and accurate as possible. After the 30 s, the object returned to a default start position, during which time participants rested for 30 s to minimize any fatigue effects. This constituted one trial. Participants performed 10 trials in each condition, with a 5-min break between each condition to minimize any fatigue effects. At the end of the four conditions, participants were provided a 10-min break, following which this entire protocol was repeated. At the end of the experimental session, a total of 20 trials per condition were obtained from each participant.

#### Dependent Measures

##### Performance

We computed three task performance metrics: tracking error (TE), precision score (P_recS_), and proximity score (P_roxS_). We defined tracking error (TE) as the minimum Euclidean distance between the participant’s position and the general space curve using linear chordal approximations. We discretized the curve into 1000 equal segments of length less than 1–10th the object’s diameter and adopted a linear approximation to identify the minimum distance between a participant’s instantaneous position (reflected by the cursor tool’s coordinates) and each line segment that constitutes the helix. We accepted the minimum distance as TE. We defined P_recS_, given by Eq. (2), as a function of TE. P_recS_ characterizes participant precision of a trial.

(2) $$P_{\text{recS}}=\frac{T\,E-\min\left(T\,E\right)}{\max\left(T\,E\right)-\min\left(T\,E\right)}$$

**FIGURE 2:**
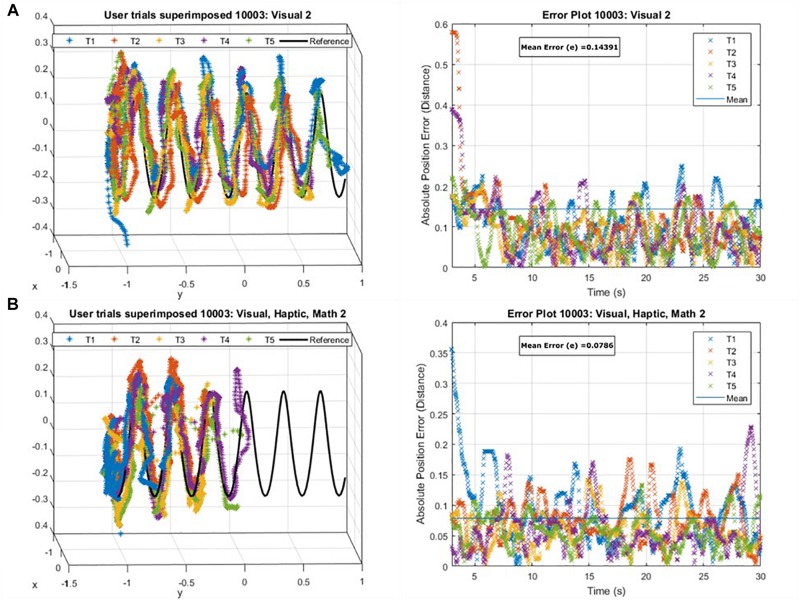
An example of user performance data superimposed onto the reference trajectory (Left) and the absolute position error as a function of time for those attempts (Right) **(A)** maximum tracking error in correspondence with low precision but high proximity scores, **(B)** minimum tracking error in correspondence to high precision but poor proximity scores for a user.

Also, we defined P_roxS_, as a function of minimum distance (*d*_*min*_) between the user’s instantaneous position and the target. This measures the participant’s task completion state based on a time history of the last n positions held by the subject for a given trial. We defined P_ro__x__S_ as:

(3) $$P_{\text{roxS}}=\frac{{\bar{d}}_{\text{n}}-\min\left({\bar{d}}_{\text{n}}\right)}{\max\left({\bar{d}}_{\text{n}}\right)-\min\left({\bar{d}}_{\text{n}}\right)}$$

where ${\bar{d}}_{\text{n}}$ is the average of *d*_*min*_ for the last n positions. Figure 2 shows an example of user performance, where, in accordance with our expectations maximum error corresponds to low precision and high proximity scores (i.e., the user rushes to the finish), while minimum error corresponds to high precision, but low proximity scores (i.e., the user traces the path carefully but is slow in doing so).

##### Perceived workload

We assessed participants’ subjective workload using paper and pencil version of the NASA-Task Load Index (NASA-TLX) to assess workload across six dimensions: mental demand, physical demand, temporal demand, performance, effort, and frustration (Hart and Staveland, 1988). We presented NASA-TLX subscales to participants post each condition and asked them to rate each subscale in increments of the low, medium, and high estimates in 21 gradations. We employed a paired comparisons procedure wherein we presented 15 pairwise combinations to participants and asked them to select the subscale from each pair that had the most effect on the workload in each experimental condition. We used an overall workload score of the NASA-TLX as a measure of perceived workload.

##### Neural activity

Functional near-infrared spectroscopy employs near infrared absorption properties of hemoglobin to infer local concentration changes of cortical oxygenated and deoxygenated hemoglobin as correlates of functional brain activity (Gateau et al., 2018). Each fNIRS measurement channel is made up of two optodes, an optical emitter (source) and a receiver (detector), with an inter-optode distance of 2.5 to 4 cm that allows near infrared to penetrate the head of an adult human to a depth of 1.5 to 2.5 cm. The difference between near infrared absorption rates for oxygenated hemoglobin (HbO) and deoxygenated hemoglobin (HbR) allows for the change in oxygen concentration to be measured (Seraglia et al., 2011).

**FIGURE 3:**
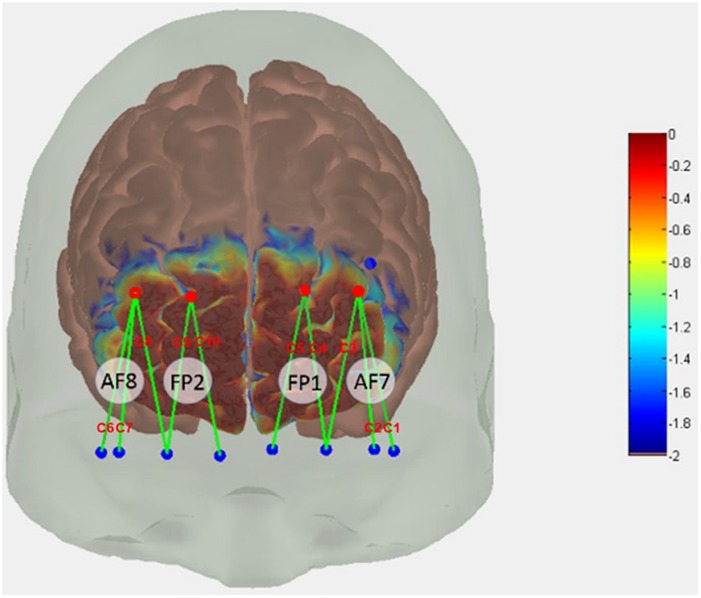
Functional near-infrared spectroscopy (fNIRS) probe design. Red dots represent sources, blue dots represent detectors. Green lines represent channels created between optodes. C1–C10 are channel labels.

In the present study, we used continuous fNIRS (Techn, Inc., Milford, MA, United States, CW6 System) to record light intensity in two wavelengths (690 and 830 nm) discharged through 4 emitters and recorded at 8 detectors to obtain hemodynamic response at a total of 10 channels based on 10/20 international EEG system (Figure 3). We converted acquired light intensities into optical densities by taking the logarithm of input signals. As recommended by Huppert et al. (2009), signal noise originating from the equipment, experiment, and physiological sources were addressed and corrected for before converting optical density into hemoglobin concentration using the modified Beer-Lambert law (Delpy et al., 1988). In order to reduce high-frequency noise, we low pass filtered the optical densities at 3 Hz. We used a spline interpolation algorithm (Scholkmann et al., 2010) to detect and correct motion artifacts that showed abrupt changes, and wavelet algorithm (Chiarelli et al., 2015) to smoothen the signals. To reduce the effects of physiological noise and slow wave drifts, we bandpass filtered motion artifact corrected signals at 0.5 ∼ 0.016 Hz. Like Cheng et al. (2012), Foy and Chapman (2018), Modi et al. (2018), and Rhee and Mehta (2018), we used a cut-off frequency of 0.5 Hz (2 s) to reduce high-frequency instrument noise and physiological (e.g., heartbeat) noise. Indeed, Zhu et al. (2019) recommend band-pass frequency of 0.01 to 0.5 Hz, to remove physiological noise and possible slow wave drift caused by the fNIRS system. Like Rhee and Mehta (2018), we used the cut-off frequency of 0.016 Hz (∼1 min) to remove slow wave drift caused by the Techen fNIRS system. Finally, we calculated HbO and HbR levels at the 10 channels using the modified Beer-Lambert law (Delpy et al., 1988). Using Atlasview (Aasted et al., 2015), we determined the location of the 12 optodes (i.e., 4 emitters and 8 detectors) that created the 10 channels.

We used HbO because it shows greater task-related changes that HbR (Rhee and Mehta, 2018). Similar to that reported by Hyodo et al. (2016) and Rhee and Mehta (2018), we first obtained task-related neural action within each trial by averaging 2 s around maximum HbO activation, from which mean HbO were computed across the 20 trials per condition. We then adopted a region of interest (ROI)-based approach (Soltanlou et al., 2018; Vanzella et al., 2019) to increase sensitivity to smaller effects (Powell et al., 2018). We defined four ROIs within the PFC – left lateral (C1 and C2), left medial (C3, C4, and C5), right lateral (C8, C9, and C10), and right medial (C6 and C7) PFC (see Figure 3). Mean HbO values from the channels in each ROI were averaged to obtain overall mean HbO per ROI per condition.

##### Neural efficiency

The neural efficiency (NE) hypothesis posits that intelligent individuals or experts efficiently utilize neural resources when engaged in a task better than less intelligent individuals or novices do (Haier et al., 1988). According to Curtin and Ayaz (2019), the NE concept can be extended to quantify the relationship between neural activity and performance, computed using Eq. (4).

(4) $$N\,E=\frac{z\,\left(p\right)-z\,\left(M\,E\right)}{\sqrt{2}}$$

where *z(P)* and *z(ME)* are z-score measures of performance and mental effort respectively. When NE = 0, performance and mental effort are balanced.

In the present study we standardized HbO levels, and the tracking error performance measure in the respective experimental conditions in order to compare the relative efficiency in those conditions. We computed the relationship between neural activation and TE in the ROIs over other performance measures, because TE is a widely used performance measure in HRI, in general, and robotic surgery, in particular (Sen et al., 2016; Enayati et al., 2018). Each standardized performance measure and standardized HbO levels were projected onto the identity line. We adopted an ROI-based approach to determine the relationship between neural activation in these regions and task performance for each of the four experimental conditions –visual feedback, visual feedback under stress, visual + haptic, visual + feedback under stress, using Eq. 5 below:

(5) $$N\,E_{\text{ij}}=\frac{z\,\left(p_{\text{i}}\right)-z\,\left(H\,b\,O_{\text{ij}}\right)}{\sqrt{2}}$$

where *i* ∈ {visual, visual + haptic}, *j* = 1…4, *P*_i_ is the tracking error performance in the *i*th feedback modality, *HbO*_ij_ is oxygenated hemoglobin level in the *jth* ROI in feedback modality *i*.

### Statistical Analyses

We conducted separate three-way mixed factors analysis of variance (ANOVAs) to test main and interactive effects of feedback (visual vs. visual + haptic), cognitive stress (no computation vs. computation), and gender (male vs. female) on the three task performance metrics. Furthermore, for each ROI, we conducted separate mixed ANOVAs on neural activity (i.e., HbO values) with gender as a between-subjects factor, and feedback (visual vs. visual + haptic) and cognitive stress (no computation vs. computation) as within-subjects factors. Finally, for each ROI we conducted separate gender × feedback × cognitive stress ANOVAs on NEs. We used Bonferroni corrections for *post hoc* comparisons and applied Greenhouse–Geisser corrections if the sphericity assumption was violated. We conducted post-analysis using Tukey’s test where needed and set the level of significance to 0.05.

## Results

### Performance

Tracking error was not significantly affected by stress, gender, or any two- or three-way interactions (all *p*’s > 0.219). There was a marginal feedback main effect, *F*(1,9) = 3.919, *p* = 0.079, *$\eta_{\text{p}}^{2}$* = 0.303, such that performance was better in the visual (*M* = 0.117, S*D* = 0.005) than visual + haptic (*M* = 0.125, *SD* = 0.008) feedback condition. Furthermore, there were no significant stress, feedback, gender, or any two- or three-way interactions on precision score (all *p*’s > 0.207). Moreover, precision score was not significantly affected by stress, gender, or two-or three-way interactions (all *p*’s > 0.166). There was a significant stress main effect, *F*(1,9) = 14.350, *p* = 0.004, *$\eta_{\text{p}}^{2}$* = 0.615, on proximity score, with performance under no stress (*M* = 0.702, *SD* = 0.032) better than under stress (*M* = 0.548, *SD* = 0.056). However, proximity score was not significantly affected by feedback, gender, or any two-or three-way interactions (all *p*’s > 0.186).

### Perceived Workload

Perceived workload, measured as overall workload score of the NASA-TLX, was not significantly affected by feedback, gender, or two- or three-way interactions (all *p*’s > 0.224). There was a significant stress main effect, *F*(1,10) = 16.932, *p* = 0.002, $\eta_{\text{p}}^{2}$ = 0.629. Participants perceived higher workload under stress (*M* = 68.368, *SD* = 3.733) than under no stress (*M* = 59.874, *SD* = 0.032).

### Neural Activity

**FIGURE 4:**
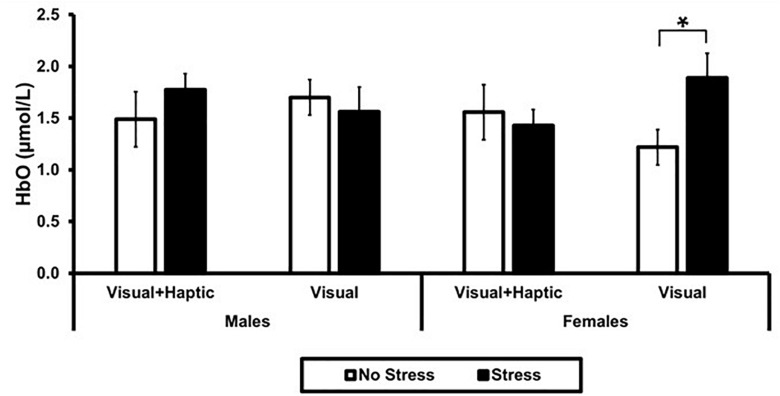
Mean HbO in right medial PFC. Error bars represent standard error. The asterisk indicates a significant difference (*p* < 0.05) between no stress and stress conditions in females.

Using ROI-based group analysis, we found that two of the four ROIs, right medial and right lateral, were significantly activated. A three-way interaction between stress, feedback and gender approached significance [*F*(1,10) = 4.905, *p* = 0.051, $\eta_{\text{p}}^{2}$ = 0.329] in the right medial PFC, and revealed a significantly higher activation in females under stress (*M* = 1.89, *SD* = 0.52) than no stress (*M* = 1.22, *SD* = 0.38) for visual feedback; *t*(10) = 2.23, *p* = 0.03 (see Figure 4). There were no main effects of stress, feedback, and gender, or any two-way interactions (all *p*’s > 0.202) in the right medial PFC.

A significant stress and gender interaction was found in the right lateral PFC; *F*(1,10) = 6.257, *p* = 0.031, $\eta_{\text{p}}^{2}$ = 0.385. Overall, there was greater activation under stress than no stress, but the effect of stress depended on the participant’s gender. Male participants demonstrated greater activation under stress (*M* = 1.67, *SD* = 0.22) than no stress (*M* = 1.22, *SD* = 0.33); *t*(10) = 2.711, *p* = 0.032. There were no main effects of feedback, stress and gender or their two-way or three-way interactions in the left lateral (all *p*’s > 0.162) and left medial (all *p*’s > 0.171).

### Neural Efficiency

There was no significant stress, feedback, gender, or any two- or three-way interactions in the left lateral (all *p*’s > 0.406) and left medial (all *p*’s > 0.171) PFC.

**FIGURE 5:**
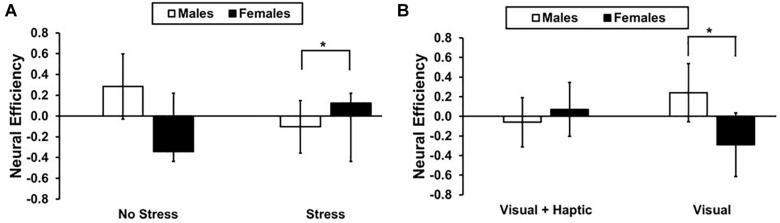
Neural efficiency for males and females in right lateral PFC **(A)** Stress and gender interaction. The asterisk indicates a significant difference (*p* < 0.05) between males and female under stress. **(B)** Feedback and gender interaction. The asterisk indicates a significant difference (*p* < 0.05) between males and female in visual feedback condition. Error bars represent standard error.

There was a significant stress and gender interaction [*F*(1,10) = 7.794, *p* = 0.021, $\eta_{\text{p}}^{2}$ = 0.464], with females (*M* = 0.124; *SD* = 0.445) exhibiting higher efficiency than males (*M* = −0.103, *SD* = 0.510) under stress in the right lateral PFC; *t*(10) = 2.262, *p* = 0.024 (see Figure 5A). Furthermore, a significant feedback and gender interaction [*F*(1,10) = 10.339, *p* = 0.011, $\eta_{\text{p}}^{2}$ = 0.535] revealed higher efficiency for males (*M* = 0.241, *SD* = 0.873) than females (*M* = −0.290, *SD* = 0.486) in the visual feedback condition; *t*(10) = 2.306, *p* = 0.021 (see Figure 5B). There were no main effects of feedback, stress and gender or their three-way interactions (all *p*’s > 0.414).

**FIGURE 6:**
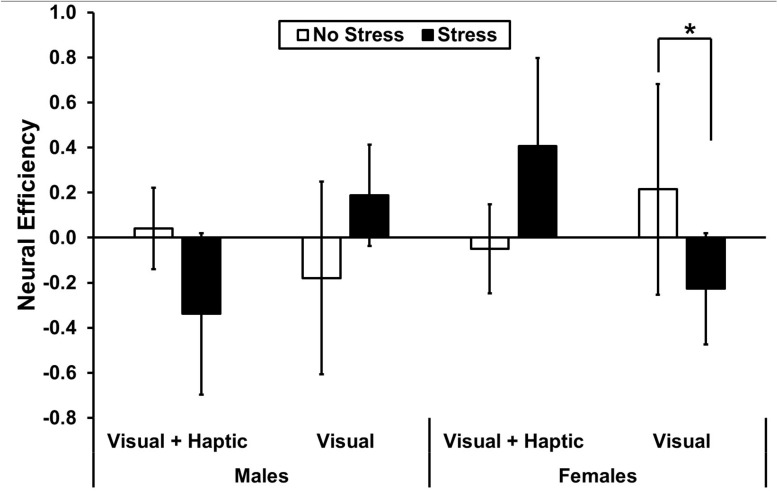
Neural efficiency in right medial PFC. The asterisk indicates a significant difference (*p* < 0.05) in neural efficiency for visual feedback under no stress and stress for females. Error bars represent standard error.

A significant three-way interaction between stress, feedback and gender [*F*(1,10) = 6.418, *p* = 0.032, $\eta_{\text{p}}^{2}$ = 0.416] in the right medial PFC revealed significantly higher neural efficiency for females under no stress (*M* = 0.215, *SD* = 0.408) than stress (*M* = −0.226, *SD* = 0.477) in the visual feedback condition; *t*(10) = 2.23, *p* = 0.03 (see Figure 6). There were no main effects or two-way interactions (all *p*’s > 0.407) in the right medial PFC.

## Discussion

The present study found that during a simulated surgical HRI task, task performance (tracking error) is lower under multiple modalities (i.e., visual + haptic) than visual only feedback, and under stress than non-stress scenarios, but remained comparable between males and females. However, we found gender differences in regions of the PFC activated under stress; males exhibited increased activation of the right lateral PFC, while females exhibited increased activation of the right medial PFC. We found that the two feedback modalities were associated with varying neural efficiencies, and these were stress- and gender-dependent. Our findings engender further investigation into effectiveness of feedback modalities on males and females under stressful conditions in HRI.

The primary task in the present study was akin to a guided motor task (Feygin et al., 2002; Sigrist et al., 2013) with concurrent feedback (visual and visual + haptic), in which task performance was measured in terms of tracking error, precision, and task completion state. Consistent with Feygin et al. (2002), Liu et al. (2006), and Yang et al. (2008), we found that task performance (tracking error) was better with visual than visual + haptic feedback. Haptic guidance in the visual + haptic feedback condition was supposed to provide additional proprioceptive cues. However, our results suggest the visual + haptic feedback modality did not improve task performance, aligning with inferences made from prior studies that combining feedback modalities does not always lead to optimal performance (Benz and Nitsch, 2017).

Higher workload is associated with increased stress (Arora et al., 2010; Klein et al., 2012) which results in degraded performance (Yu et al., 2017). In the present study, higher perceived workload in the stress condition suggests that participants did find the computational aspect of the TSST stressful. Consistent with Wetzel et al. (2006) and Arora et al. (2010) task performance (proximity score) was impaired by stress. This also corroborates with findings reported in the larger motor control literature on the impact of stress on motor performance and coordination (Mehta and Agnew, 2012; Mehta, 2015; Mehta and Rhee, 2017).

Stress results in the activation of the hypothalamic-pituitary-adrenal (HPA) axis and autonomic nervous system (Ulrich-Lai and Herman, 2009; Felmingham et al., 2012), as well as activation or deactivation of various cortical structures (Kogler et al., 2015a). The PFC, which is known to integrate information from different modalities (Fuster et al., 2000), is the most implicated cortical structure in stress response (Arnsten, 2009; Kogler et al., 2015b; McKlveen et al., 2015). It is noteworthy that traditional research on feedback modalities (e.g., McMahan et al., 2011; Koehn and Kuchenbecker, 2015; Pacchierotti et al., 2015) has focused and based conclusions on task performance measures (such as time to completion, speed, total distance traveled) and subjective responses. In the present study, we posited that operator performance is the summation of task performance and neurophysiological cost of maintaining that performance (see Equation 1), and investigated that performance may be affected by feedback, gender and a contextual factor like stress. Whereas prior studies have associated stress with increased oxygenation bilaterally within the PFC (e.g., Yang et al., 2009; Takizawa et al., 2013; Brugnera et al., 2017), others have associated stress only with right hemisphere activation (e.g., Tanida et al., 2007; Sakatani et al., 2010). We found stress to be associated with significant activation in the right lateral and right medial PFC, suggesting the right hemisphere to be dominant in the control of sympathetic activity (Brugnera et al., 2017), particularly during motor tasks (Mehta and Parasuraman, 2014). The right medial PFC has been found to play a key role in facilitating stress hormone responses through interactions with the HPA axis (Cerqueira et al., 2008). The significant change in HbO may be due to increased use of regulatory capacities (Kalia et al., 2018). By extending the analysis of stress response to different regions of the brain, we provide a better picture of how the effects of stress are integrated in the brain.

Although stress response in both genders involves activation of the HPA and sympathetic system, men and women differ in their response under stress (Wang et al., 2007; Verma et al., 2011; Kalia et al., 2018). Findings from prior studies (Kajantie and Phillips, 2006; Jaušovec and Jaušovec, 2010; Hausmann, 2017) suggest that differences in stress response between males and females may be due to hormonal effects. Earlier studies have found higher HbO concentrations in males than females during verbal working memory (Li et al., 2010) and mental arithmetic (Yang et al., 2009) tasks. It is noteworthy that males and females differ significantly during resting state – men demonstrated a higher HbO (Jaušovec and Jaušovec, 2010; Chuang and Sun, 2014). In the present study, the two-way stress and gender interaction in the right lateral PFC with a medium effect size (η*_p_^2^* = 0.385) revealed higher HbO in males than females under stress, results consistent with Li et al. (2010) and Cinciute et al. (2018). Greater HbO in males than females may have been due to increased neuronal activation or differences in brain and morphology (Ruigrok et al., 2014; Choleris et al., 2018). Aside from hormonal effects, particularly estrogen which mediates differences in the PFC’s stress sensitivity (Shansky et al., 2004), the difference in stress response between men and women in the present study may also be due to anatomical and physiological differences (Hunter, 2014). Furthermore, we found that females demonstrated higher HbO under stress than no stress in the visual feedback modality, suggesting that they recruited more neural resources to cope with stress and maintain performance (Mehta and Parasuraman, 2014; Mehta, 2016).

Generally, males outperform females in visuospatial tasks (Cutmore et al., 2000; Halpern et al., 2007; Thorson et al., 2011; Ali et al., 2015), although few studies have shown females outperform males in object rotation tasks (Silverman et al., 2007; McGivern et al., 2012). In the present study males outperformed females in the visual feedback condition, suggesting that males required relatively fewer neural resources to maintain task performance. Furthermore, in the visual feedback condition, women performed better under no stress than stress. It is evident from this study that the optimal combination of feedback modalities depends, in part, on individual characteristics (e.g., gender; Park et al., 2012), and context (e.g., stress; van Huysduynen et al., 2016; Adebiyi et al., 2017). Contrary to our expectations, the visual + haptic feedback modality did not distribute the neural cost across visual and haptic sensory modalities such that cognitive resources required for task execution will be reduced (Wickens, 2002; Sigrist et al., 2013).

Previous studies have shown that individuals vary in their response to stress, and that intra-individual variability over time may provide information about how individuals differ from each other (Sliwinski et al., 2009; Wang et al., 2012). Detection of group mean differences is enhanced when intra-individual variability is small compared to inter-individual variability (Wang et al., 2012). In the present study, the sample size was too small for meaningful analyses of individual differences. Nonetheless, we were able to detect differences in group means in the measures.

This study has some limitations. First, the task was not a pure representation of a surgical robotic task. However, given that there have been no studies that have investigated stress and gender effects, our first step was to conduct the study with a controlled robotic manipulation task. The next step is to investigate these factors with actual surgical robotics trials. Second, while the study sample size was small, this was a completely within-subject design that exhibited medium to big effect sizes. Future work that considers a larger and more diverse sample size (e.g., surgical population, age, expertise) are warranted. Third, the present study focused on a limited combination of modalities – future work should include multiple modalities with different stimulus salience parameters. Fourth, our fNIRS system covered only frontal portions of the head. Future work will utilize a whole-head fNIRS system to investigate other brain regions that may likely be recruited to preserve motor performance. Fifth, we did not record hormonal contraceptives and menstrual cycle phase for female participants. Since these are known to influence cortisol responses to stress in women, future work will investigate how they impact neural efficiencies of HRI feedback modalities under stress in women. Finally, we used perceived workload measured as the overall workload score of the NASA-TLX and known to be associated with stress (Arora et al., 2010; Klein et al., 2012), as proxy for stress. There were no physiological or subjective measures of how stressed the participants were. Future work should include measuring levels of stress, as the response may be individual specific.

## Conclusion

To our knowledge, this is the first study to investigate how gender and stress moderate the effectiveness of multimodal feedback during HRI. We posited that operator performance is a summation of task performance and neurophysiological cost of maintaining that performance. We found that while task performance was higher with visual than visual + haptic feedback, it degraded under stress. Furthermore, the two feedback modalities were found to be associated with varying hemodynamic activations and neural efficiencies, and these were stress- and gender-dependent. Our findings suggest that it is crucial to consider stress and gender differences when designing feedback in HRI.

## Data Availability

The datasets generated for this study are available on request to the corresponding author.

## Ethics Statement

This study was carried out in accordance with the recommendations of the Federal Regulations for Protection of Human Research Subjects (45 CFR 46) with written informed consent from all subjects. All subjects gave written informed consent in accordance with the Declaration of Helsinki. The protocol was approved by the Institutional Review Board at Texas A&M University.

## Author Contributions

WM and RK developed the study apparatus, conducted the data collection, performed the initial analysis, and drafted the initial manuscript. RM and SR conceptualized the study. RM analyzed the study results, and edited and revised drafted manuscript. JN analyzed the study results and revised the drafted manuscript.

## Conflict of Interest Statement

The authors declare that the research was conducted in the absence of any commercial or financial relationships that could be construed as a potential conflict of interest.
